# Body odors (even when masked) make you more emotional: behavioral and neural insights

**DOI:** 10.1038/s41598-019-41937-0

**Published:** 2019-04-02

**Authors:** Cinzia Cecchetto, Elisa Lancini, Domenica Bueti, Raffaella Ida Rumiati, Valentina Parma

**Affiliations:** 1 SISSA – International School for Advanced Studies, Neuroscience Area, Via Bonomea, 265, 34136 Trieste, Italy; 2 Institute of Psychology, University of Graz, Graz, Austria; 3 BioTechMed, Graz, Austria; 4 ANVUR - Agenzia Nazionale della Valutazione del sistema Universitario e della Ricerca, Via Ippolito Nievo, 35-00153 Roma, Italy; 5 William James Center for Research, ISPA - Instituto Universitário, Rua Jardim do Tabaco 41, 1149-041 Lisboa, Portugal; 6 Department of Clinical Neuroscience, Karolinska Institutet, Nobels väg 9, 17177 Stockholm, Sweden

## Abstract

Morality evolved within specific social contexts that are argued to shape moral choices. In turn, moral choices are hypothesized to be affected by body odors as they powerfully convey socially-relevant information. We thus investigated the neural underpinnings of the possible body odors effect on the participants’ decisions. In an fMRI study we presented to healthy individuals 64 moral dilemmas divided in incongruent (real) and congruent (fake) moral dilemmas, using different types of harm (intentional: instrumental dilemmas, or inadvertent: accidental dilemmas). Participants were required to choose deontological or utilitarian actions under the exposure to a neutral fragrance (masker) or body odors concealed by the same masker (masked body odor). Smelling the masked body odor while processing incongruent (not congruent) dilemmas activates the supramarginal gyrus, consistent with an increase in prosocial attitude. When processing accidental (not instrumental) dilemmas, smelling the masked body odor activates the angular gyrus, an area associated with the processing of people’s presence, supporting the hypothesis that body odors enhance the saliency of the social context in moral scenarios. These results suggest that masked body odors can influence moral choices by increasing the emotional experience during the decision process, and further explain how sensory unconscious biases affect human behavior.

## Introduction

Moral choices are most often explained as a result of emotional and cognitive processes^1–8^. However, morality is primarily a social phenomenon, tightly dependent on the social context. In their Relationship Regulation theory (RR), Rai and Fiske^9^ highlight the role of social context in shaping moral choices and posit that people are led by moral motives to evaluate and guide one’s own and others’ judgments and behaviors, according to moral rules developed within specific social relationships. In other words, people build a particular moral motive allowing to live in a specific social context while moral transgressions are defined as the circumvention of such specific relational prescriptions^9^. Recent empirical evidence supports this theory: for instance, participants’ moral acceptability of tradeoff scenarios can be affected by unconscious biases, such as intergroup prejudices and stereotypes, and the perception of different social groups influences the neural systems implicated in moral choices^10^.

Unconscious biases can influence moral decisions based on a variety of stimuli such as attitudes (such as dispositions towards people or places)^11^, implicit stereotypes (such as judging a person as attractive or unintelligent because is a cheerleader)^11^ or somatic reactions (such as endocrine release or psychophysical reactions)^12^. However, this line of research has not yet considered the possibility of evaluating the effects of social information transmitted via sensory subliminal cues, such as odors. Humans transfer socially-relevant information, such as age, gender, health status, sexual availability and personal predispositions, via body (or social) odors^13,14^. Furthermore, odors – including people’s odors – are everywhere and we do not necessarily realize their presence consciously^14,15^.

The idea of using olfactory stimuli to investigate moral choices is not entirely new. Landy & Goodwin^16^ argued how olfactory influences on morality are greater than those mediated by vision, the sense humans mostly rely on. Additionally, Schnall *et al*.^17^ demonstrated that the presence of a disgusting odor toughens the judgment on vignettes without moral content. Also, as we have previously shown, the subliminal exposure to a neutral odor can bias moral choices towards options characterized by harm avoidance (deontological options)^18^. Generally speaking, a harm is justified, and to some extent forgiven, if it comes as the side-effect of a moral action carrying a greater benefit compared to an intentional harm with the same outcome^19,20^. All in all, odors are able to transfer social information and their effect on moral choices seems to modulate harm avoidance.

To our knowledge, previous studies have only explored the behavioral effects of olfactory contextual stimuli on moral choices^17,18^. However, whether and how the social context might impact moral decision making when induced via sensory subliminal stimulation, and the neural underpinnings of moral choices under the exposure of masked body odors, are still unknown. A meta-analysis showed that, in absence of odor stimulation, moral (vs. non-moral) choices were found to be associated with increased activations in primarily cognition-related areas (i.e., MTG, left and right middle temporal gyrus; rMFG, right middle frontal gyrus; rIFG, right inferior frontal gyrus) and primarily emotion-related areas (i.e., cingulate gyrus, left precuneus)^21^. However, the way in which moral dilemmas are formulated modulates the competition between the fast, automatic emotional response and the slow, deliberative cognitive system. As previously shown, instrumental dilemmas (Footbridge-type dilemmas)^22^ recruit emotion-related brain areas such as medial prefrontal cortex, posterior cingulate cortex/precuneus, amygdala, and brain areas involved in “theory of mind” such as the temporoparietal junction (TPJ) and angular gyrus^1,2,4^. On the other hand, accidental dilemmas (trolley-type dilemmas) are associated with activations in neural areas involved in working memory and cognitive control, such as the dorsolateral prefrontal cortex and inferior parietal lobe^1,2,4^.

While the impact of the exposure to body odors on the neural underpinnings of moral choices still remains unexplored, we are now aware that processing body vs. common odors^23^ rely on distinct neural pathways, in line with what occurs when social information is presented through other sensory modalities (e.g., Schupp *et al*.^24^ for vision and Belin *et al*.^25^ for audition). Processing body odors recruits the occipital cortex, active when either visual stimuli or socially-relevant stimuli are cross-modally presented^26^, the angular gyrus, responsive to human body related information^27^ but also involved in social cognition and multisensory integration; and the anterior and posterior cingulate cortex, previously found implicated in emotion regulation^28,29^ and self-reflective processes^30^. What still remains to be clarified is whether these regions are also involved in the perception of human body odors during a concurrent cognitively demanding task (such as making moral decisions). If this were the case, we would expect a reduction in the activation of OFC or of the higher order areas described (e.g., posterior cingulate cortex) as a result of a reduced attention for sensory analysis, in line with the reduced activation of amygdala^31^ or piriform cortex^32,33^ observed when complex judgments are performed during odor perception.

In the present work, we hereby tested whether and how introducing a social context through masked body odors impact the behavioral and the neural correlates of moral choices. The aims of the study were the following: (1) to test whether subliminally presented body odors have a selective effect on incongruent moral dilemmas (real dilemmas) or generalize to different types of decision-making scenarios (congruent or fake dilemmas); and (2) to investigate whether and how body odors impact harm avoidance decisions. In the present functional magnetic resonance imaging (fMRI) study, participants were asked to answer to 64 moral dilemmas during the presentation of a fragrance neutral in pleasantness (masker) or to a body odor concealed by the same fragrance (masked body odor). The main dependent variable was the type of moral choice made, which could be *utilitarian*, if participants decided to execute harmful actions in order to save people, or *deontological*, if participants decided not to cause harm to not violate societal norms, even if the harm is meant for a greater good^2,19^. To explore whether the effect of the masked body odor is modulated by the dilemmatic nature of the presented scenario, half of the dilemmas were congruent, meaning that cognitive and emotional processes converged towards the same deontological action so that they were fake dilemmas, and half were incongruent dilemmas in which the two processes diverged, so they were real dilemmas^34^. Moreover, to clarify the modulation of the type of harm, half of the dilemmas were instrumental (dilemmas in which the harm is deliberate) and the other half were accidental (dilemmas in which the harm is a side effect).

We hypothesized that the presence of body odor would induce the participants to perceive the people involved in the scenario as more concrete, real. If that were the case, then participants are expected to be more prone to follow societal norms not to harm people. We anticipated this effect to be stronger than the increase of deontological answers shown when a neutral odor is presented^18^. Since it has been shown that when dealing with incongruent (compared to congruent) dilemmas, individuals were found to be more willing to provide utilitarian answers^34^, we expected such trend to be reduced in the presence of the masked body odor. Moreover, as in a previous study^18^ we observed that the presence of a neutral odor increases the number of deontological answers specifically for instrumental dilemmas, here we expected the presence of the masked body odor to result in an increment of deontological answers for such dilemmas.

With respect to the neural underpinnings, we hypothesized that the processing of incongruent (compared to the congruent) dilemmas would be associated with brain regions commonly implicated in this type of task, such as the amygdala, the ventro-medial prefrontal cortex (vmPFC^1,35^, the temporo-parietal junction^36^ and the precuneus^21^. Additionally, we predict that the presence of the body odor would favor activations in areas commonly associated with social information, including body odor processing, such as the angular gyrus, occipital cortex, and the anterior and posterior cingulate cortex^14,23^. We further hypothesized that when processing dilemmas that describe intentional harm, emotional brain areas, such as the cingulate gyrus or precuneus, would be more strongly activated. We expected that these emotional areas would be more strongly activated in the presence of the masked body odor, even when processing accidental dilemmas, usually associated with cognitive neural areas. However, given the innovative nature of this research, we had no clear predictions as to the specific neural areas to be recruited, and we therefore explore whole-brain activations with respect to this contrast.

## Materials and Methods

### Donors

Ten healthy, heterosexual males donated their body odors in two different days (age: 26.3 ± 3.6 years old (mean ± SD); range = 20–31). Male donors were chosen based on the greater intensity of their body odor axillary secretions^37^. The donors reported: (i) to be non-smokers^38^; (ii) not to have health issues or to undergo drug treatment known to be related to olfactory alterations; (iii) to have an age ranging from 18 to 35 years old. Informed written consent was obtained from each donor. Each donor agreed to follow behavioral, nutritional (i.e., no alcohol, smoking, food altering the natural body odor) and hygiene instructions throughout the collection session (adapted from)^14^. The medium of body odor collection was a t-shirt, previously washed with an odorless detergent (Liquid Detergent ECOR with no Perfume and essential oils, ECOR 27094). T-shirts were worn by donors for 12 consecutive hours during the day, right after having taken a shower using fragrance-free body wash and having dried themselves with towels washed with the same odor-free detergent used to pre-wash the t-shirts. Donors collected their body odors on separate t-shirts for each day of collection for a total of two days. Odorless plastic bags were provided to each donor to store each of their t-shirts before bringing them to the lab, the day after each collection period^23,39^. Samples were perceptually evaluated for odor contamination (e.g., alcohol, smoke, fragrance, food) and for body odor detectability by one to three trained experimenters. All samples were then stored in a −80 °C freezer to prevent sample deterioration^40^.

### Participants

The original group of participants was composed of 30 women. The rationale for testing only women is based on the evidence that women show a greater preference for social emotional stimuli^41^, also when presented in olfactory form^42^. The participants followed the same criteria as the donors, and additionally, they had to score at the 16-item Sniffin’ Sticks Identification subtest of the Sniffin’ Sticks Extended test above 10^43^ as well as presenting a regular menstrual cycle^44^.

No depression or heightened sensitivity to disgust (Disgust Scale)^45^ was revealed. Two participants were removed from the study because of possible clinical problems. The final sample included 28 healthy, heterosexual, right-handed women aged between 19 and 34 (23.7 ± 4.2 years), who were normosmic (TDI score: 13.4 ± 1.5, range = 11–16), and whose STAI state score before the task was within the normal range (STAI state score: 33.7 ± 4.3, range = 24–42). Participants were instructed to not eat or drink anything but water one hour prior to testing, and to not wear any scented products on the day of testing. The SISSA Ethics Committee approved the study, which is in accordance with the Declaration of Helsinki and an informed written consent was obtained from each participant.

### General procedure

**Figure 1:**
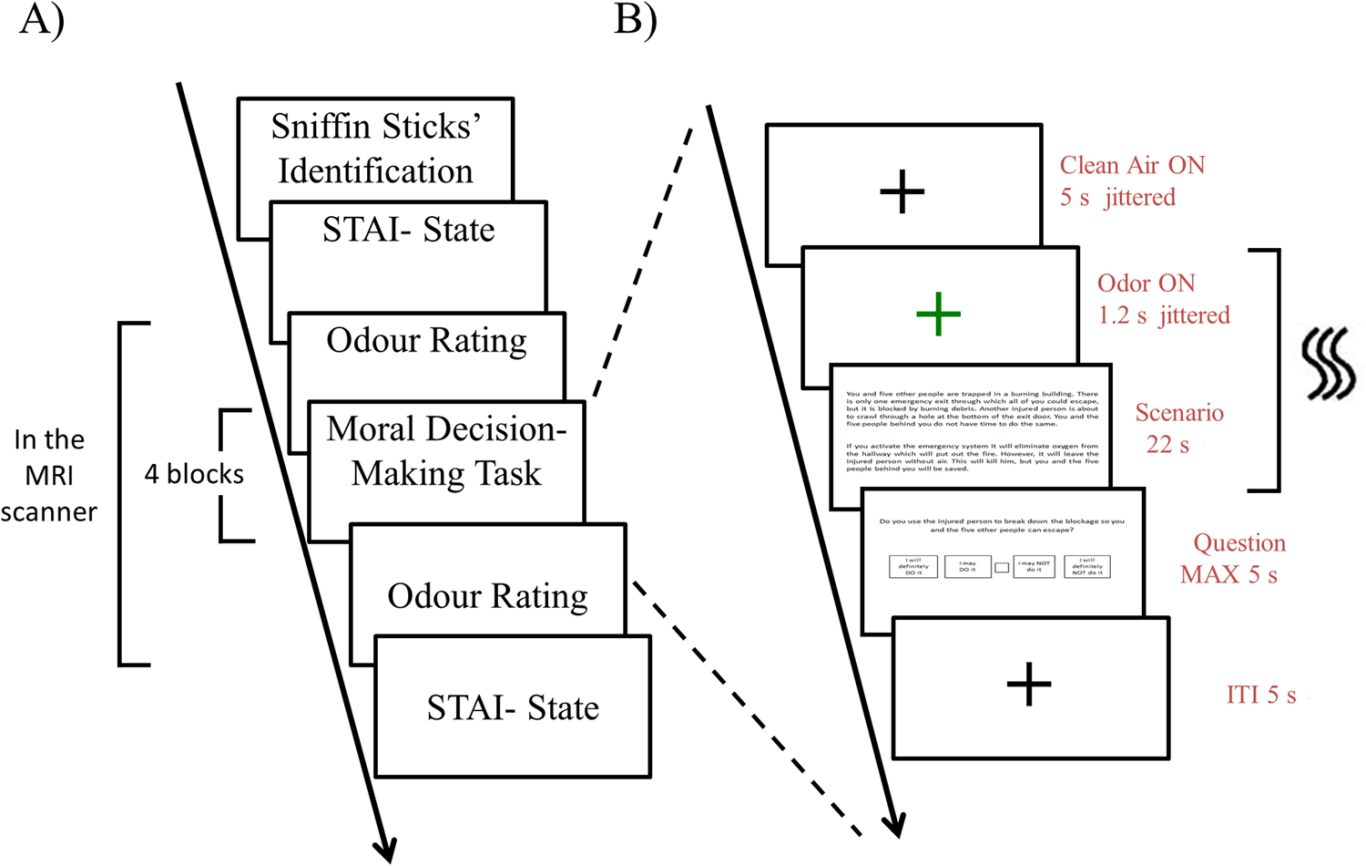
Overview of the experimental procedure. (**A**) Overview of the experiment session; (**B**) Overview of a single trial of the moral decision-making task. See Fig. S1 of the Supplementary Information for an overview of the type of moral dilemmas and odor conditions.

At the beginning of the experiment, participants were seated in a quiet room and they were instructed about the experiment. Then participants performed the odor identification test^43^ and they completed the State questionnaire of the State-Trait Anxiety Inventory (STAI-S)^46^. Anxiety state data were collected because previous literature has shown that moral choices are modulated by individual variability in anxiety^18,47,48^. To test whether the masking procedure supposed to cover the body odor produced the expected perceptual impact to the same extent across olfactory conditions, participants were asked to rate intensity, pleasantness and familiarity of the masker, masked body odor and clean air before and after the moral decision-making task. The three tasks were all performed inside the scanner in order to override the possible confounding effects of the MRI scanner setting. The procedure of the odor-rating task and of the moral decision-making task was similar to the one applied in previous study^18^ (see Supplemental Information for details about the two tasks). Then, outside the scanner, participants completed again the STAI State questionnaire^44^. See Fig. 1 for an overview of the experimental procedure.

### Odor stimuli

Two odor conditions were presented within participants. One set of dilemma alternatives (N = 32) was presented during the exposure to an emotionally neutral, rather unfamiliar odor (aka, *masker odor*; 200 μL of cedarwood oil, Sigma-Aldrich), as determined via pilot studies (see Supplementary Information of^49^ for detailed descriptions of the odor pilots) and as confirmed by previous study^18^. The masker odor was applied to equally-sized quadrants of cotton white t-shirt previously washed with the same detergent used for the t-shirts worn by the donors. The second set of dilemmas alternatives (N = 32) was presented during the exposure to the *masked body odor*. The masked body odor was prepared by including in a glass jar four donated t-shirt quadrants (supradonor) chosen from all those collected from the 10 donors and one clean t-shirt quadrant on which we applied 200 μL of masker odor^50^. The masking procedure was used to simulate the hygiene products usually used with the goal of making the paradigm more ecologically valid^51^. As customary in human body odor research^14,52–55^, each recipient smelled one supradonor stimulus across all dilemma trials, but in order to reduce the stimuli similarity^52^, the combination varied in terms of the axilla the sample came from and the day at which it was collected. The order of the two odor conditions presentations was randomized across subjects and across the four blocks of the moral decision-making task.

Odors were presented bi-rhinally in a temporally-precise, square-shaped manner using a computer-automated olfactometer^56^. A low bi-rhinal flow rate of 1.0 L/m (a total of 0.5 L/m per nostril) was used to prevent irritation of the nasal mucosa over time^56,57^. Odor stimuli were delivered directly to both participants’ nostrils from a nasal manifold, attached to the participant’s chest by means of a chest strap, connected to the olfactometer via Teflon tubing.

### Odor rating task

A green fixation cross lasting for 0.5 s preceded each odor presentation. The odor presentation lasted for 4.0 s and was accompanied by a black screen. Subsequently, a white screen was presented for 6.0 ± 0.1 s (mean ± SD) during which participants were asked in succession and in a random order to answer the following questions: “How intense was the odor you just smelled?”, “How pleasant was the odor you just smelled?”, and “How familiar was the odor you just smelled?”. During question presentation, clean air was released to minimize odor residuals^56^. Perceptual ratings for odor intensity, pleasantness, and familiarity were collected on a 10-cm computerized Visual Analogue Scale (VAS), ranging from “not at all” to “very much”. Participants were instructed to answer even if they did not perceive any odor. The odor rating task was performed inside the scanner to reduce the time of the experimental session, but without collecting functional MRI data.

### Moral decision-making task

The 4CONFiDE moral set described in Cecchetto *et al*.^20^ was reshaped for this study to include congruent and incongruent dilemmas. A total of 64 dilemmas was presented, 32 congruent and 32 incongruent. Furthermore, half of the congruent and incongruent dilemmas were accidental and the remaining instrumental. Each dilemma type was presented in 16 alternative versions to allow for the presentation of the same factor combination in both odor conditions. The order of presentation of the dilemmas was randomized across participants to exclude any presentation order effects on moral decision-making (see Fig. S1 in the Supplementary Information for a visualization of the features of the dilemma set).

Each dilemma was presented on two subsequent screens. The first screen described the scenario, in which a danger threatens to kill a group of persons, and a hypothetical action would save these people but cause the death of another person. The second screen presented the question *Do you…[action verb] so that…?* Participants had to choose between four options: “I certainly do it”, “I do it”, “I do not do it”, and “I certainly do not do it”. The first two choices are held to be utilitarian, as they maximize overall utility (i.e., saving more lives), whereas the latter two were non-utilitarian (deontological).

Before starting the moral decision-making task, participants performed two practice trials. An Italian version of the instructions suggested by Christensen *et al*.^19^ and previously used in Cecchetto *et al*.^20^ was administered.

See Fig. 1 for an overview of the moral decision-making task. Each trial began with a black cross that was displayed for 5.0 ± 0.3 s. Then, a green cross was presented for 1.2 ± 0.2 s and the odor delivery started. Subsequently, the scenario was presented for 22.0 s. The scenario presentation was combined with the odor presentation. Afterwards, the question slide was presented together with the releasing of clean air to minimize odor residuals^56^. The four choices were displayed below the question. Participants had maximum 5.0 s to answer. After the answer a black cross was presented for 5.0 s.

The 64 dilemmas were divided into four blocks that corresponded to four scanning runs. During each block, 16 trials balanced for moral dilemmas types and odor conditions, were presented in randomized order. Participants were allowed to take a short break at the end of each run while lying in the scanner. Dilemmas were presented using a black font color (font: Calibri, size: 24) against a white background. Stimulus presentation was delivered with E-prime 2.0 software (Psychology Software Tools, Pittsburgh, PA).

### Behavioral data analysis

Frequency analysis was performed on the four response options to see whether the number of each option changed based on the odor condition. Since no significant differences were found among the four response options in relation to odour condition, we collapsed them for the subsequent analyses.

Behavioral data were analyzed with linear mixed-effects models (LMMs)^58^ using R (version 2.10.1; http://www.r-project.org/) and in particular using the *lme* function (*nlme* package; https://cran.r-project.org/web/packages/nlme/nlme.pdf) for continuous variables and the *glmer* function (*lme4* package; http://cran.r-project.org/web/packages/lme4/index.html) for binary variables (deontological or utilitarian answer). To account for individual differences (e.g., some people are more “deontological” than others), participants were included in the models as *random* factors. To avoid a warning of non-convergence, an optimizer (bobyqa) was applied^59^. Results with and without the optimizer are not significantly different (https://github.com/lme4/lme4/blob/master/misc/notes/release_notes.md). Estimates on the choice between utilitarian and deontological responses were based on an adaptive Gaussian Hermite approximation of the likelihood with 10 integration points. For odor ratings, models with odor and session were tested. For moral choice, two models were performed: the first included odor, as the main variable of interest of our analysis, and congruency. The second model included odor and intentionality and it was performed considering only incongruent dilemmas.

Outliers in reaction times were determined by means of the outliers-labelling rule^60^. From a sample of 1792, 127 trials were removed for no response (N = 127/1792, 7.08%), and 43 trials were removed because of extremely long choice reaction times (>2.6 s; N = 43/1665, 2.58%; mean of reaction times is 820.8 ± 507.2 s). Conditions have equivalent final samples of trials (Masker odor = 810, Masked Body odor = 812; X^2^_1_ = 0.0025, p = 0.96).

### MRI data acquisition and pre-processing

A 3 Tesla Philips Achieva whole-body MR Scanner at the University Hospital of Udine (ASUI Udine, Italy), equipped with an 8-channel head coil, was used for MRI scanning. Head movement was minimized through cushioning within the coil. Functional volumes were obtained using a whole-head T2^*^-weighted echoplanar image (EPI) sequence (repetition time [TR] = 2.5 s, echo time = 35 ms, flip angle = 90°, 28 transverse axial slices with interleaved acquisition, 3.50 × 3.59 × 4.00 mm^3^ voxel resolution, field of view = 230 × 230 mm^2^, acquisition matrix = 68 × 62, SENSE factors: 2 in the anterior–posterior direction). The number of volumes acquired varied for each participant and run given the task duration based on participants’ reaction times (mean number of volumes per run = 260 ± 4.6, range = 153–270). Anatomical images were acquired during the final odor rating task as 180 T1-weighted images (0.75 mm slice thickness). Stimuli were viewed through VisuaStim Goggles system (Resonance Technology) mounted to the head coil, which was adjusted on each participant’s vision. Responses were made and recorded through one MR-compatible response pads (Lumitouch, Lightwave Medical Industries, Coldswitch technologies, Richmond, CA) using the right hand. To minimize influences of breathing effects, participants were instructed and trained to maintain a constant and normal breathing rate. Due to technical problems, images from the first session of one participant and the second session of another participant were removed from the analysis.

Data were analyzed with SPM12 (Wellcome Trust Centre for Neuroimaging, London, UK). All functional volumes were spatially realigned to the first volume, slice- time corrected, segmented in gray matter, white matter and cerebrospinal fluid tissues, spatially normalized to the standard EPI template, and smoothed using a Gaussian kernel with full width at half maximum (FWHM) of 8 mm^3^_._ Movement-related variance was analyzed using the Art toolbox (www.nitrc.org/projects/artifact_detect). For each run, outlier scans were identified based on the TR-to-TR composite motion more than 2 mm and/or considering whether the scan-to-scan global BOLD signal normalized to z-scores deviated from mean more than z = 3. The time-points identified as outliers were regressed out as separate nuisance covariates in the first-level design matrix. All participants displayed a percentage of outlier scan inferior to the cutoff (25%), therefore no one was excluded from the analyses and all trials were retained.

### fMRI data analysis

Two separated fMRI data analyses were carried out: in the first analysis, odor conditions and congruency of dilemmas were considered to explore whether the effect of masked body odor was modulated by the dilemmatic nature of the presented scenario; in the second analysis, which was performed only on incongruent dilemmas, odor conditions and intentionality as the type of dilemmas were considered to investigate the effects of masker body odor on the processing of different types of harm.

Statistical analyses were performed using a general linear model (GLM) approach. In the first-level analysis, data were analyzed separately for each participant. In each trial, four events were modelled: the presentation of clean air, of an odor, of the scenario combined with an odor and, of the slide including the question. The duration of each of these events was set to 0 except for the scenario presentation, which was set to a fixed time of 22.0 s. The combination of these four event types with dilemma congruency (congruent vs incongruent) or dilemma intentionality (accidental vs instrumental) and the two odor conditions (masker vs masked body odor) led to a total of 16 regressors for each run. The six motion parameters were also included as regressors of no interest in the design matrix. All regressors were convolved with a canonical hemodynamic response function. Low-frequency signal drifts were filtered using a cutoff period of 128.0 s. As a next step, at the individual level, contrast parameters were estimated for all the 16 regressors of interest, averaged across the four runs. Subsequently, at the second-level analysis, 4 contrast images of the event scenario presentations from the combination odor with congruency or intentionality of each participant were submitted to a flexible factorial design, with subject as random factor, odor conditions and congruency or intentionality as fixed factors, to assess neural activations of the dilemma processing during the exposure to the odor. Later, the 4 contrast images were entered to linear contrasts of the repeated measure ANOVA with two within-subject factors to investigate main effects and interactions. To identify the neuronal substrates of single odor condition or single dilemma type, simple main effects (i.e., [masker odor – masked body odor] for each odor condition and each dilemma type separately) were analyzed. To investigate whether odor conditions affect neural activity related to moral dilemma processing, we performed a dilemma type (i.e. congruent/incongruent or accidental/instrumental) by odor condition (masker odor/masked body odor) interaction at group level. Moreover, to clarify whether the neural underpinnings involved in the masker body odor effects for one dilemma type (i.e. incongruent or accidental) are shared by the opposing dilemma type (i.e. congruent or instrumental), exclusive and inclusive conjunction analyses were performed between the neural areas recruited for the interactions [odor × congruency or intentionality].

Finally, in order to investigate the relationship between brain activations and moral choices, the mean beta values of the activated clusters were extracted using the REX toolbox (Department of Brain and Cognitive Sciences, Massachusetts Institute of Technology, MA) and simple correlation analyses were performed with the percentage of utilitarian responses.

Whole-brain analyses were thresholded at p < 0.05, family-wise error (FWE) cluster-level corrected for multiple comparisons across the whole brain. The AAL2 toolbox^61,62^ was used to guide the labelling of the activated clusters.

## Results

### Masked body odor and masker are perceptually similar

**Figure 2:**
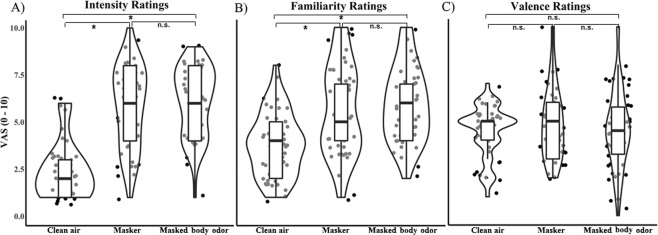
Distribution of participants’ odor ratings. The black dots represent single data points, whereas the box-plot represents the interquartile range of each distribution, with the thick black horizontal bar corresponding to the median. Each box-plot is surrounded by a violin plot representing the smoothed distribution of data. Significant differences (p < 0.05) are indicated with a star.

We first tested whether the masking procedure applied to cover the masked body odor had the expected perceptual impact and rendered the olfactory conditions equivalent in their basic perceptual dimensions. The LMM on intensity ratings (clean air: 2.45 ± 0.13 points; masker: 5.83 ± 0.18 points; masked body odor: 6.10 ± 0.16 points; see Fig. 2A and Table 1) revealed that both the masker and the masked body odor were perceived as significantly more intense than clean air (*p* < 0.001; reference factor: clean air), but no significant difference was found between the masker and the masked body odor (*p* = 0.48; reference factor: masker). A difference emerged when looking at the effect of session (pre moral decision-making task: 5.23 ± 0.21 points; post moral decision-making task: 4.56 ± 0.20 points; *p* = 0.024): odors were rated as less intense during the second session compared to the first session suggesting that participants might have adapted during the task (Dalton, 2000).

**Table 1:** Summary of linear mixed effects model on intensity, familiarity and pleasantness odor ratings.

Intensity Ratings	β	SE	t value	p value	95% CI	
*Fixed effects*					Lower	Upper
*Intercept*	6.18	0.32	19.20	**<0.001**	5.546	6.807
Clean Air	−3.31	0.35	−9.28	**<0.001**	−4.016	−2.615
Masked body odor	0.24	0.34	0.70	0.484	−0.432	0.914
Session (Post)	−0.66	0.29	−2.28	**0.024**	−1.233	−0.093
**Familiarity Ratings**						
*Fixed effects*						
*Intercept*	5.76	0.37	15.61	**<0.001**	5.035	6.480
Clean Air	−1.85	0.33	−5.61	**<0.001**	−2.502	−1.207
Masked body odor	0.27	0.33	0.83	0.410	−0.370	0.910
Session (Post)	−0.27	0.27	−0.99	0.323	−0.814	0.267
**Pleasantness Ratings**						
*Fixed effects*						
*Intercept*	5.05	0.32	15.55	**<0.001**	4.416	5.689
Clean Air	−0.47	0.31	−1.51	0.132	−1.079	0.138
Masked body odor	−0.38	0.30	−1.26	0.210	−0.976	0.212
Session (Post)	−0.20	0.25	−0.78	0.436	−0.695	0.298

*Note: β* = estimate; SE = standard error; *95% CI* = confidence interval. Significant p values are reported in bold. Table shows model with masker condition set as reference.

The LMM on familiarity ratings (clean air: 3.78 ± 0.14 points; masker: 5.61 ± 0.20 points; masked body odor: 5.91 ± 0.17 points; see Fig. 2B and Table 1) showed that both the masker and the masked body odor were perceived as significantly more familiar than clean air (*p* < 0.001; reference factor: clean air), but no significant difference was found between the masker and the masked body odor (*p* = 0.40; reference factor: masker). No significant differences were found between the ratings performed before and after the task (*p* = 0.32; reference factor: pre).

The LMM on pleasantness ratings (clean air: 4.48 ± 0.12 points; masker: 4.89 ± 0.16 points; masked body odor: 4.58 ± 0.18 points; Fig. 2C) showed no significant differences across the three odor conditions. Moreover, no significant differences were found between sessions. Please, refer to Table 1 for descriptive data.

### State anxiety is increased at the end of the task

A Wilcoxon test (W = 148801, *p* < 0.0001) determined that participants’ state anxiety was increased at the end of the task (34.36 ± 6.45 points, range = 22–48) as compared to its beginning (33.67 ± 4.33 points, range = 24–42; see Fig. S2 in the Supplementary Information).

### Irrespective of odor condition, incongruent dilemmas produce more utilitarian responses

First, the model including odor conditions, congruency and the interaction between them was performed (see Table 2 for descriptive data of single parameters). There was a significant effect of congruency on moral choice: the likelihood of choosing the utilitarian option increased when dilemmas were incongruent (z = 10.08, *p* < 0.001). In other words, when cognitive and emotional processes diverge (real dilemmas), more utilitarian answers are produced than when cognitive and emotional processes converge (fake dilemmas). No significant effects were found for the main effect of odor condition or for the interaction odor × congruency.

**Table 2:** Summary of the linear mixed effects model on moral choices with odor and congruency as fixed factors.

Moral choices	β	SE	z value	p value	βexp	95% CI	
*Fixed effects*						Lower	Upper
*Intercept*	−1.19	0.18	−6.37	**<0.001**	0.302	0.209	0.436
Masked body odor	0.25	0.16	1.50	0.13	1.282	0.926	1.775
Incongruency (*Incongruent*)	1.65	0.16	10.08	**<0.001**	5.223	3.788	7.202
Masked body odor * Incongruency (*Incongruent*)	−0.32	0.22	−1.42	0.15	0.726	0.466	1.129

*Note: β* = estimate; SE = standard error; β exp = exponential of β coefficient; *95% CI* = confidence interval. Significant p values are reported in bold. Table shows model with deontological choice and masker odor condition set as references. The contrast condition from the reference for categorical factors is reported in italic in brackets.

### Irrespective of odor condition, accidental dilemmas produce more utilitarian responses

Considering the results of the previous model, we tested the effect of odor conditions, intentionality and the interaction between these two factors on incongruent dilemmas only (see Table 3 for descriptive data of single parameters). A significant effect of intentionality emerged (z = −0.43, *p* < 0.001): in incongruent dilemmas, the likelihood of choosing the utilitarian option increased when dilemmas were accidental (vs instrumental). The odor condition, alone or in interaction, did not affect the type of moral choice made.

**Table 3:** Summary of the linear mixed effects model on moral choices with odor and intentionality as fixed factors, performed on incongruent dilemmas.

Moral choices	β	SE	z value	p value	βexp	95% CI	
*Fixed effects*						Lower	Upper
*Intercept*	0.004	0.17	0.02	0.98	1.003	0.717	1.405
Masked body odor	0.24	0.15	1.58	0.11	1.268	0.945	1.704
Intentionality (*Instrumental*)	−0.67	0.15	−0.43	**<0.001**	0.512	0.380	0.690
Masked body odor * Intentionality (*Instrumental*)	−0.34	0.22	−1.56	0.12	0.714	0.468	1.090

*Note: β* = estimate; SE = standard error; β exp = exponential of β coefficient; *95% CI* = confidence interval. Significant p values are reported in bold. Table shows model with utilitarian choice and masker odor condition set as references. The contrast condition from the reference for categorical factors is reported in italic in brackets.

### fMRI brain activations

#### Areas involved in moral cognition are selectively activated by incongruent dilemmas

**Figure 3:**
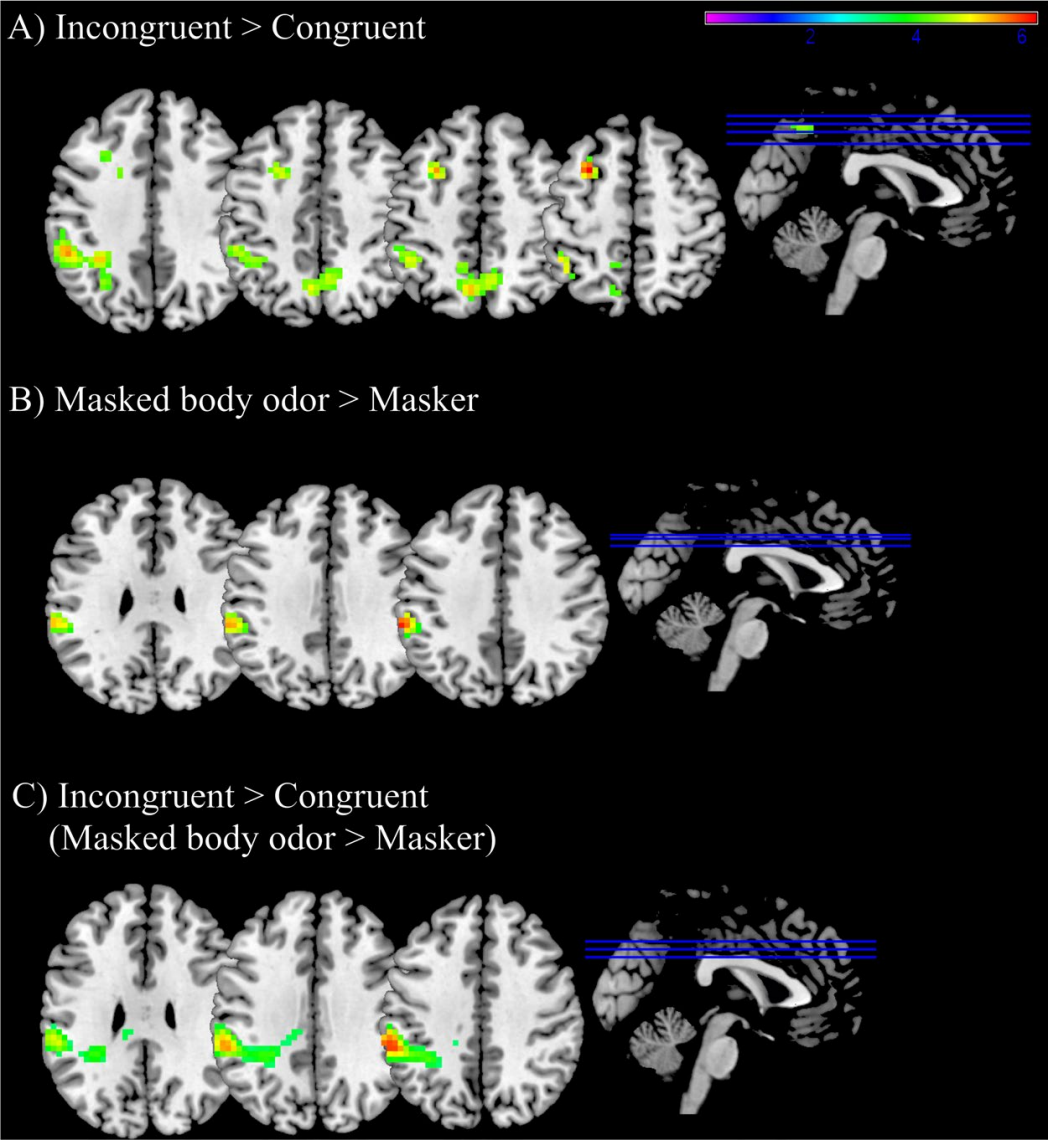
Brain activation maps showing significant cluster of activations for (**A**) Incongruent >Congruent: significant activations in the left middle frontal gyrus, left inferior parietal gyrus and bilateral precuneus; (**B**) Masked body odor >Masker: significant activations in the left supramarginal gyrus; (**C**) Incongruent (masked body odor >masker) >Congruent (masked body odor >masker): significant activations in the left supramarginal gyrus. Statistical maps are derived with a threshold of *p* < 0.05 FWE corrected and superimposed on a standard T1 template. Color scale represents t statistics. Image labels: L = left, R = right.

The processing of real dilemmas (contrast incongruent vs congruent dilemmas) revealed activations in the left middle frontal gyrus, left inferior parietal gyrus and bilateral precuneus (see Table 4 and Fig. 3A). The correlation analysis performed between beta values and percentage of utilitarian answers did not show significant results. No significant activations emerged when considering the processing of fake dilemmas (congruent vs incongruent dilemmas).

**Table 4:** Brain regions exhibiting significant differential activity for the main effects, interactions and conjunction analysis of congruency and odor conditions.

Brain region	Side	Cluster size	peak MNI coordinates			*p* (FWE-corr)	T	Z score
			x	y	z			
**Incongruent >Congruent**								
Middle frontal gyrus	L	57	−27	11	56	0.037	4.98	4.64
Middle frontal gyrus	L		−31	21	39		3.41	3.29
Inferior parietal gyrus	L	177	−52	−42	39	<0.001	4.41	4.16
Inferior parietal gyrus	L		−34	−46	39		4.17	3.96
Precuneus	L	78	−6	−67	49	0.012	4.21	4
Precuneus	L		−13	−49	53		3.81	3.65
Precuneus	R		5	−60	49		3.8	3.64
**Masked body odor >Masker**								
Supramarginal gyrus	L	72	−59	−35	42	0.017	5.39	4.97
Supramarginal gyrus	L		−62	−32	28		4.6	4.32
**Masker >Masked body odor**								
Calcarine cortex	L	259	−10	−91	−11	<0.001	4.37	4.13
Middle occipital gyrus	L		−17	−91	7		4.08	3.88
Calcarine cortex	R		8	−88	7		4.08	3.88
Precuneus	R	164	1	−53	28	<0.001	4.14	3.94
Lingual gyrus	L		−20	−53	4		3.97	3.79
Posterior cingulum	L		−6	−49	32		3.81	3.65
**Incongruent (masked body odor) >Congruent (masker)**								
Inferior parietal gyrus	L	244	−59	−35	42	<0.001	6.26	5.64
Inferior parietal gyrus	L		−45	−46	56		4.11	3.91
Inferior parietal gyrus	L		−27	−42	35		4.03	3.83
**Congruent (masker) >Incongruent (masked body odor)**								
Calcarine cortex	L	109	−10	−95	−7	0.001	4.11	3.91
Calcarine cortex	R		5	−91	0		4.05	3.86
Calcarine cortex	L		−6	−95	7		3.91	3.73
**Incongruent (masked body odor >masker) >Congruent (masked body odor >masker)**								
Supramarginal gyrus	L	233	−59	−35	42	<0.001	6.26	5.64
Supramarginal gyrus	L		−45	−46	56		4.11	3.91

*Note:* Anatomical labels follow the nomenclature of the Automated Anatomical Labelling (AAL2). Peak locations are expressed in MNI coordinates. Voxelwise threshold, *p* < 0.001. FWE cluster level corrected *p* < 0.05.

#### Activation in visual areas tracks the utilitarian responses to incongruent dilemmas when exposed to the masker odor only

The presence of the masked body odor (vs the masker odor) during the presentation of both incongruent and congruent dilemmas was accompanied by activations in the left supramarginal gyrus (see Table 4 and Fig. 3B). In contrast, the presence of the masker odor (vs the masked body odor) activates the bilateral calcarine cortex, the left middle occipital gyrus, the right precuneus, the left lingual gyrus and the left posterior cingulum (see Table 4). The beta values extracted from the cluster including the bilateral calcarine cortex and the left middle occipital gyrus significantly correlate with the number of utilitarian responses to incongruent dilemmas when exposed to the masker odor (*r* = 0.48, *p* = 0.009). No other significant correlation between behavioral responses and neural activations emerged.

#### The masked body odor during incongruent dilemmas is associated with activations in the left supramarginal gyrus

To identify whether the brain regions that are active components in the masked body odor effect for the incongruent dilemmas are shared also for the masked body odor effect in congruent dilemmas, exclusion and inclusion conjunction analyses were performed between the areas recruited for the interaction “masked body odor and incongruent dilemmas” and for the interaction “masked body odor and congruent dilemmas”. The exclusion conjunction analysis for [incongruent (masked body odor >masker) >congruent dilemmas (masked body odor >masker)] showed that the left supramarginal gyrus was significantly recruited only when the masked body odor was presented during the processing of incongruent dilemmas (see Table 4 and Fig. 3C). No significant correlations were found between the extracted beta values and percentage of utilitarian answers in this contrast. The inclusion conjunction analysis and the opposite exclusion conjunction analysis did not reveal any significant results.

#### Emotional areas are involved in instrumental vs accidental incongruent dilemmas

**Figure 4:**
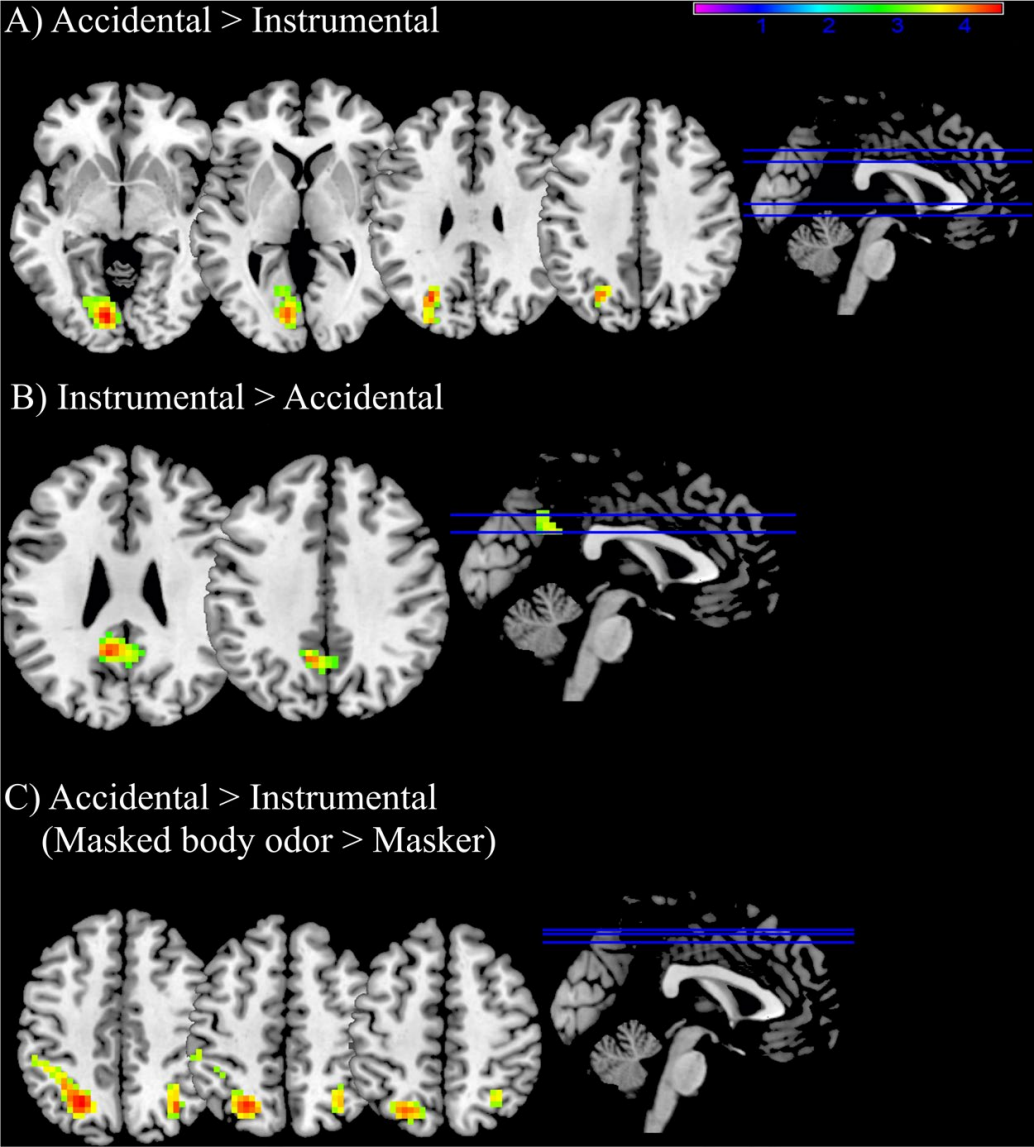
Brain activation maps showing significant cluster of activations for (**A**) Accidental >Instrumental: significant activations in the left lingual gyrus, left fusiform gyrus, the left inferior occipital gyrus and the left middle occipital gyrus; (**B**) Instrumental >Accidental: significant activations in the bilateral precuneus; (**C**) Accidental (masked body odor >masker) >Instrumental (masked body odor >masker; significant): significant activations in the left superior and inferior parietal gyrus and in the right angular gyrus. Statistical maps are derived with a threshold of *p* < 0.05 FWE corrected and superimposed on a standard T1 template (Coronal and sagittal views are displayed). Color scale represents t statistics. Image labels: L = left, R = right.

To evaluate the effect of intentionality, only incongruent dilemmas were considered. Processing accidental (vs instrumental) dilemmas significantly activated the left lingual gyrus, left fusiform gyrus, the left inferior occipital gyrus and the left middle occipital gyrus (see Table 5 and Fig. 4A), whereas processing instrumental (vs accidental dilemmas) was related to significant activation in the bilateral precuneus (see Table 5 and Fig. 4B). No significant correlations with behavioral responses were retrieved.

**Table 5:** Brain regions exhibiting significant differential activity for the main effects, interactions and conjunction analysis of intentionality and odor conditions.

Brain region	Side	Cluster size	peak MNI coordinates			p (FWE-corr)	T	Z score
			x	y	z			
**Accidental >Instrumental**								
Lingual gyrus	L	165	−10	−81	−4	<0.001	4.74	4.44
Fusiform gyrus	L		−24	−74	−7		3.95	3.77
Inferior occipital gyrus	L		−24	−95	−11		3.68	3.53
Middle occipital gyrus	L	163	−27	−70	32	<0.001	4.57	4.3
Middle occipital gyrus	L		−34	−88	14		4.29	4.07
Middle occipital gyrus	L		−27	−88	25		4.27	4.05
**Instrumental >Accidental**								
Precuneus	L	88	−10	−53	32	0.006	5	4.66
Precuneus	R		5	−56	32		3.92	3.74
**Accidental (masker odor) >Instrumental (masked body odor)**								
Calcarine	L	1252	−6	−88	7	<0.001	5.43	5.01
Lingual gyrus	L		−6	−84	−7		5.36	4.94
Calcarine	L		−20	−63	7		5.16	4.79
**Accidental (masked body odor) >Instrumental (masker odor)**								
superior parietal gyrus	L	141	−24	−63	46	<0.001	4.52	4.25
Inferior parietal gyrus	L		−34	−53	46		4.03	3.84
Inferior parietal gyrus	L		−45	−42	42		3.81	3.65
Angular gyrus	R	56	33	−67	46	0.032	4.22	4
**Instrumental (masker odor) >Accidental (masked body odor)**								
Precuneus	L	257	−10	−53	32	<0.001	7.1	6.25
Precuneus	R		1	−53	28		6.46	5.79
Angular gyrus	L	106	−48	−63	32	0.002	5.09	4.73
**Accidental (masked body odor >masker)** >**Instrumental (masked body odor >masker)**								
Superior parietal gyrus	L	140	−24	−63	46	0.001	4.52	4.25
Inferior parietal gyrus	L		−34	−53	46		4.03	3.84
Inferior parietal gyrus	L		−45	−42	42		3.81	3.65
Angular gyrus	R	56	33	−67	46	0.032	4.22	4

*Note:* Anatomical labels follow the nomenclature of the Automated Anatomical Labelling (AAL2). Peak locations are expressed in MNI coordinates. Voxelwise threshold, *p* < 0.001. FWE cluster level corrected *p* < 0.05.

#### The masked body odor during accidental dilemmas is associated with activations in the left parietal and right angular gyri

To clarify whether the brain regions that are active components in the masked body odor effect for the accidental dilemmas are shared also for the masked body odor effect in instrumental dilemmas, conjunction analyses were performed between the neural areas recruited for the interaction “masked body odor and accidental dilemmas” and for the interaction “masked body odor and instrumental dilemmas”. The exclusive conjunction analysis for accidental (masked body odor >masker) >instrumental (masked body odor >masker) showed significant activations in the left superior and inferior parietal gyrus and in the right angular gyrus. The inclusive conjunction analysis and the opposite exclusion conjunction analysis did not reveal any significant results suggesting that the masked body odor modulated only the processing of accidental dilemmas (see Table 5 and Fig. 4C). The correlation analysis between the beta values extracted and the percentage of utilitarian responses did not reveal any significant results.

## Discussion

Previous research suggests that moral rules are developed within specific social-relational contexts that, in turn, play a critical role in shaping moral choices^9,10^. As human body odors are powerful messengers for socially-relevant information^63^, able to modulate the behavior and neural processing of the receiver^13,14,23,39,53–55^, we hypothesized that body odors might affect moral choices through the modulation of the perceived social context (i.e., by inducing the perception of the real presence of a person). With this in mind, we asked participants to decide their course of action to moral scenarios while exposed to a neutral fragrance (masker) or to a body odor hidden by the same masker odor (masked body odor). The analysis of the neural correlates revealed that the exposure to the masked body odor: a) modulates the activity in the brain areas involved in the processing of incongruent (real) dilemmas, but not in those involved in the processing of congruent (fake) dilemmas; and b) increases the activations in areas processing sensory and emotional information when incongruent accidental dilemmas are presented.

In our study, we investigated whether masked body odors influence any decision-making task or whether the influence is specific to moral dilemmas, as we had anticipated. The analysis we performed revealed that the masked body odors moderate the neural responses only related to incongruent (but not congruent) dilemmas, increasing the involvement of the left supramarginal gyrus. While presented with a real (incongruent) moral dilemma, the participants immediately experience a negative emotional reaction at the thought of provoking harm: the final decision will be deontological providing that this emotional reaction is sufficiently influential, and that participants have limited time and cognitive resources to make their decision. On the other hand, if participants have enough time, motivation and cognitive resources, they will have the possibility to engage in cognitive deliberation about costs and benefits, in which case the emotional response may be overshadowed, resulting in an utilitarian response to the dilemma^2,34^. The information of the masked body odor might interfere with this conflict enhancing the neural pathways that promote prosocial behavior^64^, therefore emphasizing the emotional processing of the sensory information, and facilitate the emergence of deontological responses. This multisensory integration of the social and sensory information provided by the masked body odor, and the emotional information provided by the moral dilemmas involve the left supramarginal gyrus, close to the angular gyrus^1^, one of the neural areas previously found to be associated with the processing of body odors^2,3^. The supramarginal gyrus has been often considered as part of the temporo-parietal junction (TPJ) - a neural area typically associated with self-awareness and body-related information processing^4,5^ and, as such, often involved in tasks of theory of mind^5,6^, empathy for pain^7^ and in the perception of anxiety body odors^8^. Importantly, these aspects become relevant when considering a body odor in the context of moral dilemmas. Indeed, the centrality of the left supramarginal gyrus in multisensory integration processes has recently been supported in a study that identifies this brain area as an important node for the olfactory-visual processing^9^.

Since in the fake (congruent) dilemmas there is no conflict between emotional and cognitive aspects of the decision (i.e., the benefits do not balance the costs), the social information about the presence of a person becomes irrelevant for the decision itself.

Moreover, in the present study, we clarified whether the masked body odor effect is modulated by the type of harm, being it deliberate (instrumental dilemmas) or an inadvertent effect (accidental dilemmas). Previous studies showed that the accidental harm is judged as being more morally acceptable, it receives higher percentage of utilitarian answers, and it engages lower emotional reactions compared to intentional harming^4,64^. Our results are in line with this literature: instrumental dilemmas presented higher percentage of deontological answers and recruited neural areas involved in emotional processing (e.g., precuneus) when compared to accidental dilemmas. Interestingly, the masked body odor seems to moderate the processing of the accidental dilemmas by enhancing the activation of the angular gyrus, which is usually associated with social cognition, multisensory integration and “theory of mind”^27^, and the inferior parietal gyrus, which is important for self-other discrimination^65^. This result seems to support our hypothesis that the presence of a body odor can induce the participant to perceive the social context of the dilemmas as more concrete, as if the odor signaled the presence of a real person, and not just of a hypothetical context. The reason why the masked body odor seems to selectively affect the processing of the accidental and not of the instrumental dilemmas may be due to the higher emotional involvement of the instrumental dilemmas, which prevents the participants to consider the additional emotional information provided by the odor.

The present fMRI data replicate and extend previous findings concerning the neural networks recruited by social odor processing^14,15,23^. Besides replicating the enrollment of the left supramarginal gyrus, as discussed above, we also showed major activity in the left hemisphere areas. This result, in line with previous studies^23,66^, supports the hypothesis that olfactory-mediated affective processes are lateralized in the left hemisphere^67^.

In our study, the body odor was masked by a neutral odor. This masker was applied to simulate the hygiene products usually used to cover the natural body odors we produce and to make the paradigm more ecologically valid^51^. Additionally, it allowed studying the effects of the body odor when they are unconsciously perceived. As seen in previous work^18^, odor effects can emerge irrespective of perceiving the presence of an odor; moreover, masking the body odor limited the inter-individual differences in odor intensity and pleasantness. Such differences can significantly affect decisions, as it seemed in the previous cases when intensity and pleasantness differences across odor conditions were evident^17,18^. Here we succeeded in making these conditions perceptually similar for intensity, pleasantness and familiarity, removing the possible confounding effect of these factors on the differences in the moral decisions.

To our knowledge, this is the first study that tests the effects of masked body odors on the neural underpinning of moral decision-making. The present study has some limitations for which future studies are necessary. First, it was designed around a moral decision-making paradigm based on the presentation of moral dilemmas, which felt dilemmatic as the participants’ anxiety levels raised at the end of the task. The use of this sort of dilemmas has been previously criticized (e.g.)^55,64,68^: (i) dilemmas are described in lengthily written texts, which increase the time needed by the participants to process each stimulus; (ii) to make dilemmas credible they cannot be repeated; (iii) the conceptual factors cannot be analyzed separately, but have to be intermingled in each dilemma. These aspects reduce the possibility to present large numbers of trials, therefore limiting the power of the study. To overcome these issues, we have used here a standardized, culturally-equivalent moral set, specifically designed for imaging experiments, that shows high consistency across the different dilemmas^20^. Moreover, to increase the power of our observations, we based the investigation on a theoretically-motivated interest for one conceptual factor (Intentionality). Despite these efforts, the behavioral analysis failed to reveal any significant mean effects of the odor conditions or significant interactions with odor and dilemma congruency or intentionality. One proposed explanation is that the dilemmas were designed to simultaneously assess also other conceptual factors, such as personal force, benefit recipient and evitability. It is for future studies to clarify whether the masked body odor elicits different effects on moral choices when different conceptual factors are considered (see for example)^69^. Second, the participants’ respiratory patterns were not recorded and incorporated in the fMRI data processing. Although this is common practice in many olfactory neuroimaging studies^70,71^, we invite future studies to investigate whether the breathing patterns to human body odor can have an impact on the moral decisions made. Third, only one common odor (cedarwood oil) has been used as masker, and the results cannot be generalizable to all common odors. Fourth, future studies should also increase the sample size to allow the comparison of masked body odors effects in women and men and evaluate potential sex-related effects. Lastly, it would be interesting if future investigations would be extended to clinical populations with a deficit in emotion processing, such as patients with lesions in the ventromedial prefrontal cortex^72^, or non-clinical populations with emotional deficiencies, such as those with a lack of empathy or with high levels of alexithymia^73,74^, to examine whether the masked body odor effects on moral decision-making can overcome the usual tendency in this population to give higher percentage of utilitarian answers^74^.

To conclude, the value of these results is highlighted by the consideration that most of the moral decisions, from everyday choices to choices that we are forced to make under unexpected circumstances, are made in the presence of other people. Starting from the theory proposed by Rai and Fiske^9^, which advanced the hypothesis that actions and outcomes should be considered in the context of specific social relationships, indeed any action - including violence and impure acts - can be perceived as morally acceptable depending on the social relationships it takes place in^9^, body odors were used as a means for triggering the social context and for making the social norms more salient. Our results indicate that body odors could effectively mediate moral decisions, possibly increasing the emotional experience during the decision process, and this effect is possible even when the perceiver cannot appreciate the presence of the body odors. Moreover, the current results suggest that, as Cikara *et al*.^10^ posited, the context in which the decisions are made is relevant for understanding which decision is made.

## Supplementary information

Supplemental material

## Data Availability

The datasets generated and/or analyzed during the current study are available from the corresponding author on reasonable request.
