# Supplementary material for: Body odors (even when masked) make you more emotional: behavioral and neural insights
Source: Sci Rep. 2019 Apr 2;9:5489. doi: 10.1038/s41598-019-41937-0 (PMC6445102; doi:10.1038/s41598-019-41937-0)

**Body odors (even when masked) make you more emotional: behavioral and neural insights**

Cinzia Cecchetto^1,2,3*^, Elisa Lancini^1^, Domenica Bueti^1^, Raffaella Ida Rumiati^1, 4^ and Valentina Parma^1,5,6^

^1^ SISSA – International School for Advanced Studies, Neuroscience Area, Via Bonomea, 265, 34136 Trieste, Italy

^2^ Institute of Psychology, University of Graz, Graz, Austria

^3^ BioTechMed, Graz, Austria

^4^ANVUR - Agenzia Nazionale della Valutazione del sistema Universitario e della Ricerca, Via Ippolito Nievo, 35 - 00153 Roma, Italy

^5^ William James Center for Research, ISPA - Instituto Universitário, Rua Jardim do Tabaco 41, 1149-041 Lisboa, Portugal

^6^ Department of Clinical Neuroscience, Karolinska Institutet, Nobels väg 9, 17177, Stockholm, Sweden

**Supplemental Information**

**Materials and Methods**

**Figure S1.** Overview of types of moral dilemmas and their distributions over the odor conditions. The order of presentation of the dilemmas was randomized across participants to exclude any presentation order effects on moral decision-making.


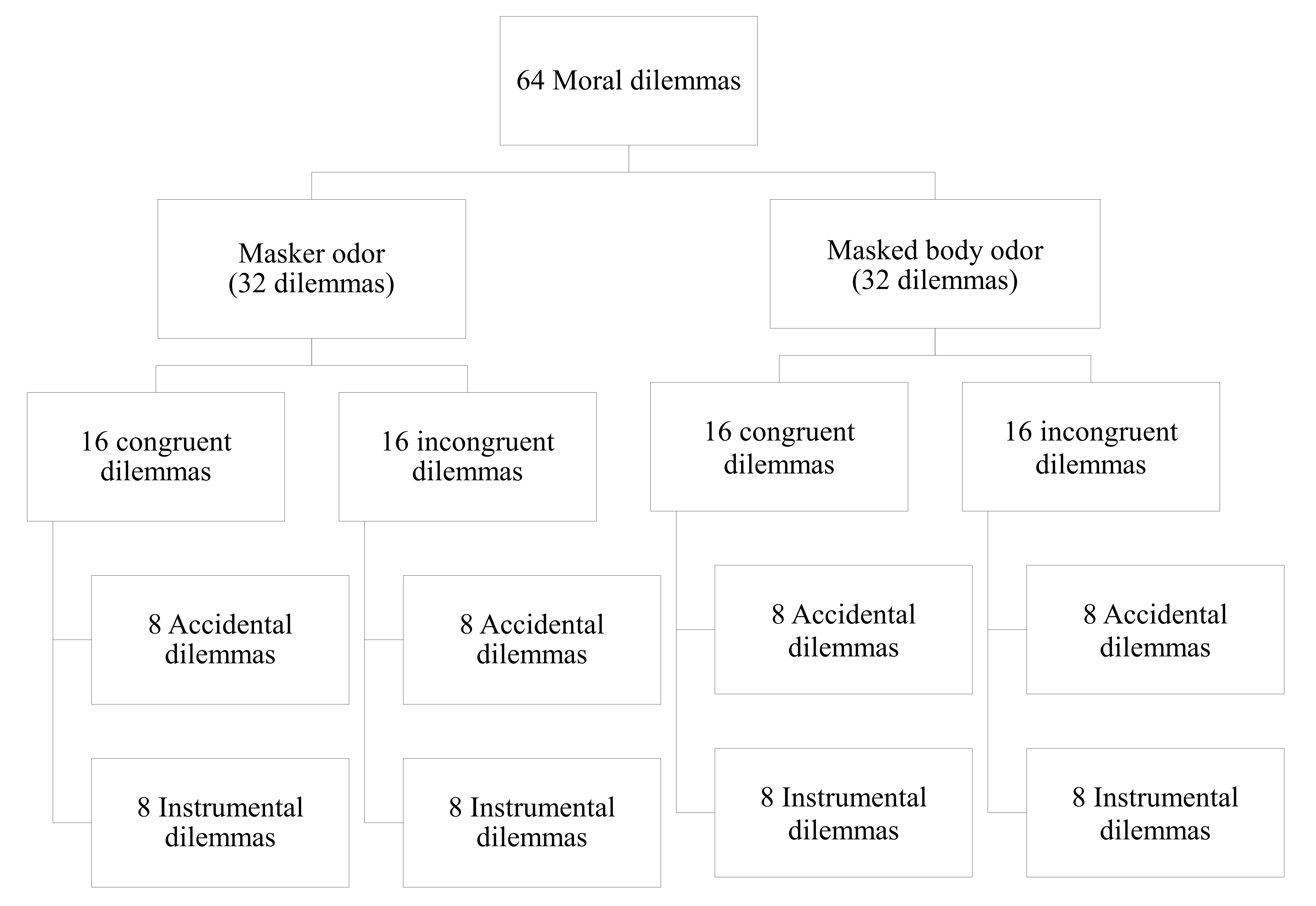


**Results**

**Figure S2.** Non-linear relationship between STAI state (STAI-S) pre and post the moral decision-making task


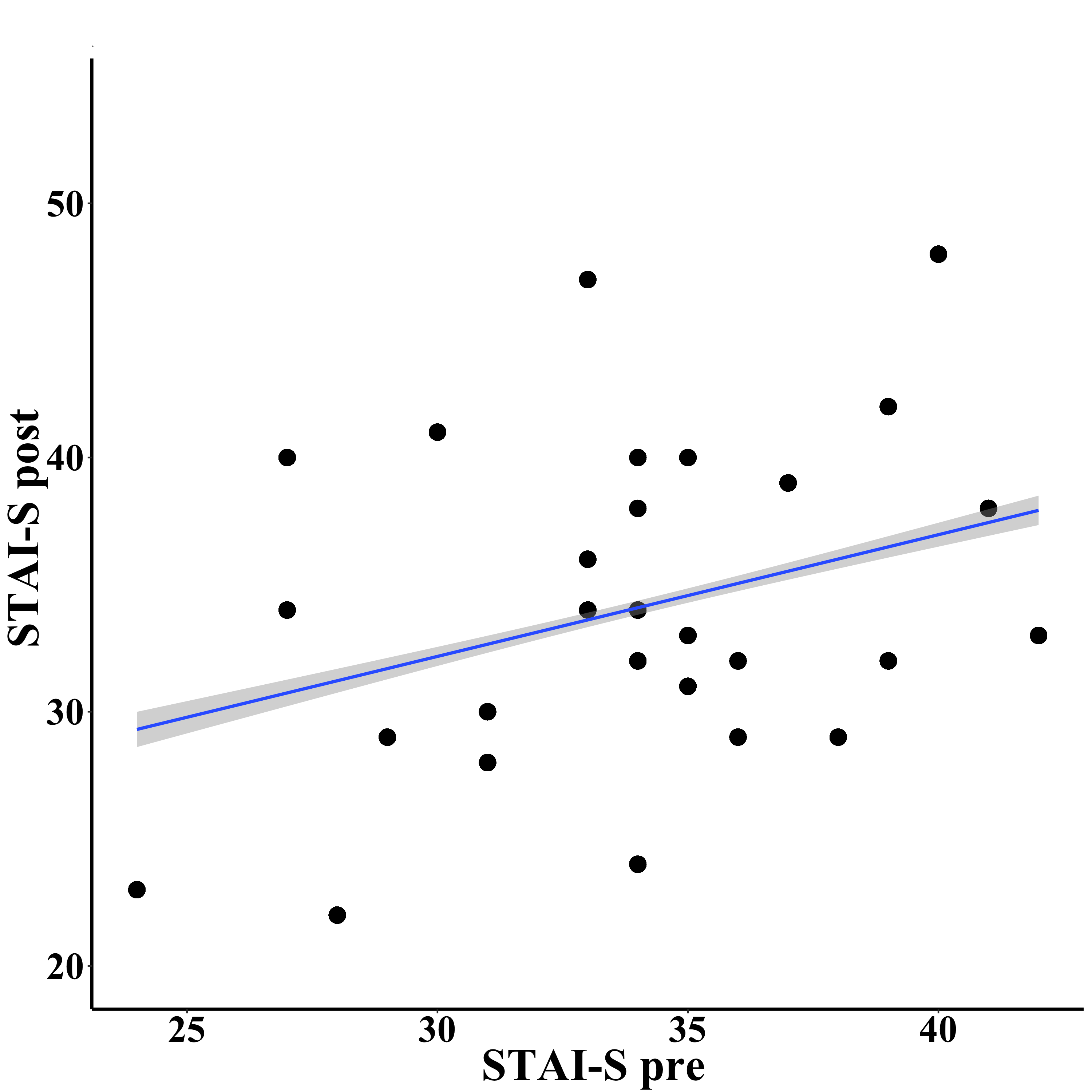

Supplement: Supplementary file 1 — Supplemental material [file 41598_2019_41937_MOESM1_ESM.docx]
